# Effects of photobiomodulation therapy on upper limb lymphedema secondary to breast cancer: a systematic review and meta-analysis

**DOI:** 10.3389/fonc.2026.1802643

**Published:** 2026-03-23

**Authors:** Chenjie Qian, Zixin He, Chunhua Liu, Yuan Fang, Yongfei Zheng, Zunjing Zhang, Zhi Ye

**Affiliations:** 1 Rehabilitation Center, The People’s Hospital of Cangnan, Wenzhou, Zhejiang, China; 2 Graduate Joint Training Base, Zhejiang Chinese Medical University, Hangzhou, Zhejiang, China; 3 Department of Obstetrics and Gynecology, Lishui Hospital of Traditional Chinese Medicine Affiliated to Zhejiang University of Chinese Medicine, Lishui, Zhejiang, China

**Keywords:** breast cancer, function, lymphoedema, pain, photobiomodulation

## Abstract

**Background:**

Breast cancer–related lymphedema (BCRL) is a common complication that impairs function and quality of life. The effectiveness of photobiomodulation (PBM) therapy remains unclear, necessitating a systematic review and meta-analysis.

**Methods:**

This review followed the PRISMA guidelines and was registered in PROSPERO (CRD420261296197). PubMed, CENTRAL, and Embase were systematically searched for randomized controlled trials (RCTs) evaluating PBM therapy for BCRL up to January 20, 2026. Outcomes of interest included upper limb volume, circumference, grip strength, and pain. Pooled effects were synthesized using appropriate meta-analytic models and reported as mean differences with 95% confidence intervals. Risk of bias, methodological quality, and certainty of evidence were assessed using RoB 2, the PEDro scale, and GRADE, respectively.

**Results:**

Nine RCTs involving 312 participants with BCRL were included. Compared with control conditions, PBM therapy resulted in significant reductions in affected limb volume (SMD = −0.78, 95% CI −1.03 to −0.54; I² = 22%) and limb circumference (MD = −3.61 cm, 95% CI −4.85 to −2.38; I² = 44%). Functional outcomes also improved, with increased grip strength (MD = 1.72 kg, 95% CI 1.07 to 2.37; I² = 14%) and reduced pain intensity (MD = −0.29, 95% CI −0.52 to −0.05; I² = 0%). Overall risk of bias was low to moderate, with some concerns about allocation concealment; evidence certainty was mostly moderate by GRADE.

**Conclusions:**

PBM therapy is associated with significant improvements in limb volume, circumference, grip strength, and pain in patients with BCRL. Despite these favorable findings, the overall certainty of evidence is moderate, and safety conclusions are limited by sparse adverse-event reporting. Larger, well-designed RCTs are needed to confirm efficacy and optimize treatment parameters.

## 1 Introduction

In recent years, the global burden of breast cancer (BC) has continued to increase, making it the most frequently diagnosed malignancy among women. In 2020, approximately 2.3 million new cases were reported worldwide, with projections indicating that annual incidence will exceed 3 million by 2040 (1). BC remains one of the leading causes of cancer-related mortality in women (2). Advances in surgery, radiotherapy, chemotherapy, and systemic therapies have substantially improved survival; however, prolonged survival has also led to a growing prevalence of treatment-related long-term complications. Among these, breast cancer–related lymphedema (BCRL) has emerged as a major concern, significantly impairing physical function and quality of life in survivors (3).

BCRL commonly occurs after breast cancer surgery, axillary lymph node removal, and radiotherapy, which damage lymphatic pathways and compromise lymph transport, resulting in the accumulation of protein-rich fluid in the interstitial space (4). Long-term follow-up studies indicate that more than 20% of breast cancer survivors develop upper limb lymphedema, with risk increasing over time (5). Clinically, BCRL manifests as persistent limb swelling and pain, often accompanied by restricted mobility, muscle weakness, sensory changes, and recurrent infections (6). In advanced stages, progressive fibrosis and joint impairment may develop. These physical limitations are frequently associated with psychological burden, reduced social participation, and diminished work capacity, leading to substantial declines in quality of life. Currently, BCRL lacks a definitive curative treatment and is managed primarily with conservative approaches (7). Complete decongestive therapy (CDT), comprising manual lymphatic drainage, compression, skin care, and exercise, remains the standard of care (8). While CDT can reduce edema and alleviate symptoms, its benefits depend heavily on sustained adherence and prolonged treatment (9). Practical challenges, including treatment complexity, discomfort, skin-related adverse effects, and activity restriction, often limit long-term compliance (10). Furthermore, existing therapies focus largely on symptom management and offer limited ability to restore lymphatic function or reverse established fibrosis, leaving a considerable proportion of patients with persistent or progressive disease.

Given the limitations of conventional conservative therapies in terms of long-term adherence, symptom recurrence, and reversal of fibrosis, increasing attention has been directed toward safe, non-invasive, and repeatable adjunctive interventions. Photobiomodulation therapy (PBM) has gradually been introduced into the clinical management of breast BCRL and has shown potential benefits in reducing limb swelling and symptom burden in some randomized controlled studies (11, 12). From a biological perspective, PBM acts on tissues through red or near-infrared light, influencing mitochondrial energy metabolism and related signaling pathways, thereby modulating microcirculation, inflammatory responses, and tissue repair processes (13, 14). In the context of lymphedema pathophysiology, PBM may promote lymphangiogenesis, enhance lymphatic drainage, and attenuate chronic inflammation and fibrosis, thereby improving tissue compliance and reducing fluid accumulation (15, 16). In addition, its immunomodulatory, analgesic, and soft-tissue reparative effects provide a theoretical basis for improvements in pain, functional limitation, and disability among patients with BCRL. However, existing clinical studies are limited by small sample sizes and substantial heterogeneity in treatment parameters and outcome measures, which restricts the consistency and generalizability of the evidence.

Therefore, this study aimed to systematically evaluate the effects of PBM therapy on limb swelling, function, and pain in patients with BCRL through a systematic review and meta-analysis, and to assess the certainty of the available evidence.

## 2 Methods

The study protocol was prospectively registered in the PROSPERO database (CRD420261296197), and the review methodology adhered to PRISMA recommendations.

### 2.1 Search strategy

The review process consisted of three sequential phases: identification of records, eligibility screening, and final study inclusion. Randomized controlled trials (RCTs) were retrieved from PubMed, Embase, and the Cochrane Central Register of Controlled Trials (CENTRAL) from inception to January 20, 2026. Only English-language publications were eligible. Searches were structured around three domains: BCRL, PBM-related terms (including PBM and laser-based terminology), and RCT identifiers. A combination of controlled vocabulary and free-text terms was applied, with truncation used when appropriate. To ensure comprehensive coverage, reference lists of included studies were examined manually, and authors of conference abstracts were contacted when additional information was required. Full search details are presented in **Supplementary Table 1**.

### 2.2 Inclusion and exclusion criteria

Eligibility criteria were established using the PICOS framework. Studies were eligible if they included patients diagnosed with BCRL, without restrictions on age or disease duration. ①Interventions involved PBM therapy, applied alone or alongside standard care; ②Control groups received comparable background treatment without PBM, including sham procedures, usual care, conventional rehabilitation, or no additional intervention; ③Studies were required to report quantitative lymphedema-related outcomes before and after intervention, with at least one of the following measures: limb circumference, limb volume, pain, or quality of life. Only RCTs published in English were included.

Studies were excluded if they met any of the following criteria: (1) non-original publications, including letters, case reports, conference abstracts, reviews, or commentaries; (2) insufficient data to calculate effect sizes with 95% confidence intervals; or (3) failure to report efficacy outcomes, or inclusion of overlapping or duplicate datasets.

### 2.3 Study selection and data extraction

After duplicate records were removed using EndNote (Clarivate Analytics), two reviewers (C.Q. and Z.H.) independently screened all studies based on predefined eligibility criteria. Screening involved title and abstract review followed by full-text assessment to confirm eligibility and identify incomplete information. Disagreements were resolved by consensus, with a third reviewer (Z.Z.) consulted when required.

Two reviewers independently extracted data using a standardized data extraction form. Information collected included study characteristics (title, authors, and year of publication), participant characteristics (diagnostic criteria and age), sample size, and study design. Details of PBM therapy were recorded, including treatment modality and parameters (wavelength, dose, and irradiance), as well as intervention protocols (treatment duration, frequency, intervention period, and total number of sessions). Outcome measures and corresponding summary statistics (means and standard deviations before and after intervention) were extracted. The primary outcomes were limb volume, limb circumference, grip strength, and pain. In addition, adverse events were systematically sought and extracted from all eligible trials and descriptively summarized to assess safety; when adverse events were not reported, this was recorded as “not reported” rather than assumed absent.

### 2.4 Certainty of evidence assessment

Evidence certainty for the primary outcomes was evaluated using the GRADE methodology implemented in GRADEpro GDT. The assessment considered five dimensions that may reduce confidence in the results, including methodological limitations, heterogeneity, indirectness, imprecision, and potential reporting bias. RCTs were regarded as providing high-level evidence at baseline and were downgraded if concerns were identified in any domain. Certainty ratings and key findings were summarized in a GRADE Summary of Findings table.

### 2.5 Sensitivity analyses

Sensitivity analyses were conducted by sequentially removing one study at a time to examine its impact on the pooled results. Robustness was further tested by excluding crossover trials that contributed only first-phase change-score data.

### 2.6 Data synthesis and statistical analysis

Statistical analyses were conducted using RevMan (version 5.4). Effect sizes were derived from group sample sizes and post-intervention means with corresponding standard deviations. Continuous outcomes were pooled as mean differences (MD) or standardized mean differences (SMD) with 95% confidence intervals, depending on the measurement scales used. Results were presented graphically using forest plots, and statistical significance was defined as a two-sided p-value < 0.05.

For trials with more than two arms, when a single control group was shared by two eligible PBM intervention arms, we included the intervention arms as two separate comparisons. To avoid double-counting the shared control participants and artificially inflating precision, we split the control-group sample size equally across the comparisons (with group means and standard deviations unchanged), following Cochrane Handbook guidance (17).

Between-study variability was evaluated using Cochran’s Q test and the I² statistic. Fixed-effects models were applied when outcomes were measured on the same scale and heterogeneity was low (I² < 50%); otherwise, random-effects models were used. Random-effects models were also applied in sensitivity analyses to account for potential clinical and methodological differences across studies. Owing to the limited number of included trials, publication bias was not formally assessed, as funnel plots and related tests are not recommended when fewer than 10 studies are available according to the Cochrane Handbook (17).

We performed time-stratified subgroup analyses (approximately 2, 4, 8, and 12 weeks) for each outcome. Subgroup analyses by PBM parameter ranges or overall treatment duration were not feasible due to insufficient data and inconsistent parameter reporting.

## 3 Results

### 3.1 Study identification and selection

**Figure 1:**
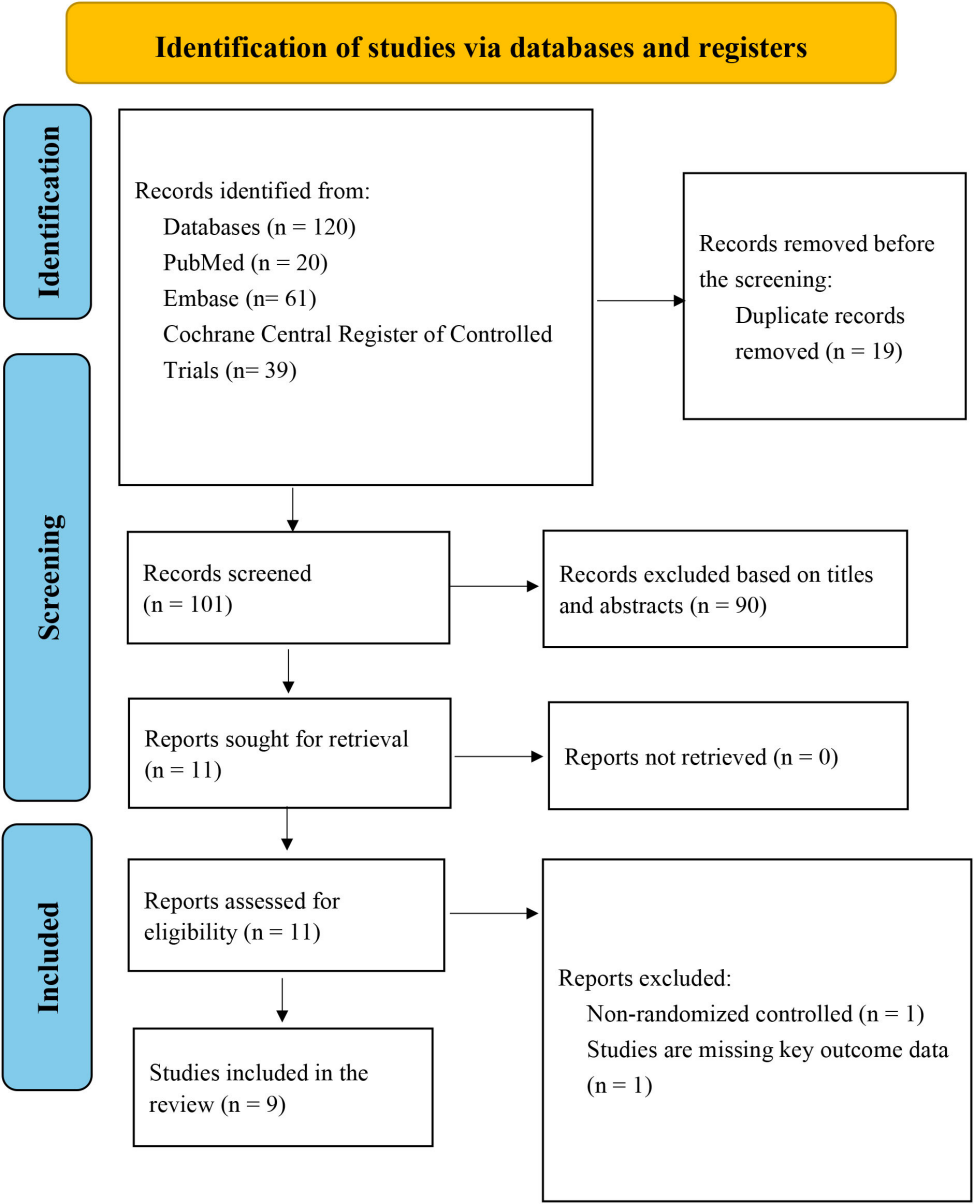
PRISMA flow diagram of study identification, screening, and inclusion.

The study selection process comprised four stages. A total of 120 records were identified through database searches conducted from inception to January 20, 2026. After the removal of 19 duplicate records by two reviewers (C.Q. and Z.H.), 101 records remained for screening. Title and abstract screening excluded 90 records that did not meet the eligibility criteria. Full-text assessment was then performed for 11 articles, of which one non-randomized study and one study lacking primary outcome data were excluded. Ultimately, nine studies met the inclusion criteria and were included in the meta-analysis (**Figure 1**).

Disagreements arising during study selection were resolved by discussion, with a third reviewer (C.L.) consulted when consensus could not be reached.

### 3.2 Study characteristics

The main characteristics of the included studies are summarized in **Table 1**. All publications were written in English and involved women aged 18 years or older with clinically diagnosed BCRL, evaluating the clinical effects of PBM. The RCTs were published between 2003 and 2023, with sample sizes ranging from 8 to 50 participants (11, 12, 18–24). All studies assessed changes in limb circumference or volume; five also reported pain outcomes, and three measured grip strength. Treatment effects were primarily compared with conventional conservative care (e.g., manual lymphatic drainage) or sham laser controls. Follow-up duration varied across studies, most commonly ranging from 1 to 3 months, with longer follow-up in some trials. Baseline edema severity was variably reported; two trials provided baseline excess limb volume (affected minus unaffected), whereas the remaining studies did not report this metric (20, 23).

**Table 1:** Characteristics of the included studies.

Study	Country	Design	Cancer stage /duration			PBM group						Control group			Outcomes	Adverse effect	Measure ment timepoint
				Sample size	Age (year)		Baseline excess limb volume (mL)	Intervention (PBM)	Treatment parameters	Treatment protocol	Stimulation sites	Sample size	Age (year)	Intervention			
Ahmed Omar MT, et al. (2011)	Egypt	RCT, 2 arms	Stages II or III breast cancer	25	54.76 ± 3.33		Not reported	Ga-As laser (Pagani IR27/4; RianCorp Pty Ltd., Australia)	904 nm (infrared, Ga-As); 5 mW; spot size 0.2 cm²; pulsed (50 ns); max 2800 Hz; 1.5 J/cm²	2 min/point at 10 points; 20 min/session; 3 sessions/week for 12 weeks (36 sessions)	Antecubital fossa (3 points); axilla/axillary lymph nodes (7 points)	25	53.36 ± 3.56	Sham laser	Limb, strength, circumference Handgrip Shoulder ROM	Not reported	4, 8, 12 weeks
Baxter GD, Liu L, et al. (2018)	New Zealand	RCT, 2 arms	Stages II or III breast cancer	9	57.90 ± 9.60		Not reported	LLLT (PBM) – LightForce EX (LTS-1500, 2012)	Solid-state laser diode; wavelength 980/810 nm (80:20); output power 500 mW; power density 100 mW/cm²; spot size 5 cm²; energy density 6 J/cm²; energy per point 30 J/cm²	60 s/point at 10 points; total radiant energy 300 J/session; 2 sessions/week; 12 sessions total	10 points along the affected arm from the axilla to the wrist	8	64.30 ± 11.10	Conventional therapy	Limb circumference Pain, Heaviness	One case of cellulitis; no other serious Adverse events	8, 12 weeks
Carati CJ, Anderson SN, et al. (2003)	Australia	RCT, 2 arms	>= 3 months	33	63.00± 2.00		888 ± 108	LLLT (PBM) – RianCorp LTU- 904H	Pulsed Ga-As laser; wavelength 904 nm; average output 5 mW; spot size 0.2 cm²; Class 1 laser; contact mode	Treatment delivered by skin contact; parameters measured at the start of each session	Affected upper limb	28	65.00± 2.00	Sham laser	Limb volume Quality of life	Not reported	4, 8, 12 weeks
Kaviani A, Fateh M, et al. (2006)	Iran	RCT, 2 arms	>= 3 months	4	53.70 ± 9.80		Not reported	LLLT (PBM) – Ga–As diode laser (Mustang-024, Russia)	Wavelength 890 nm (Ga–As); pulsed mode; output power 10 W; frequency 3000 Hz; pulse width 130 ns; emission power 4 mJ/s; spot size 0.7 cm²; energy per point 1 J; energy density 1.5 J/cm²; non-contact mode (1 cm distance)	5 points/session; 3sessions/week for 3 weeks; after an 8-week interval, the same protocol was repeated for another 3 weeks (total 18 sessions)	Axillary region (5 points)	4	48.70 ± 12.50	Sham laser	Limb circumference Pain	Not reported	4, 8, 12 weeks
Kozanoglu E, Gokcen N, et al. (2022)	Turkey	RCT, 2 arms	Stages II or III breast cancer	21	46-66		Not reported	LLLT (PBM) – (Electronica Pagani IR27/4)	Ga–As laser; wavelength 904 nm; frequency 2800 Hz; energy density 1.5 J/cm²	20 min/session; 5 sessions/week for 4 weeks (20 sessions)	Antecubital fossa (3 points); axilla (7 points)	21	49-62	Sham active	Limb, Pain circumference Handgrip, strength	Not reported	12 weeks
Lau RW, Cheing GL. (2009)	China	RCT, 2 arms	Stages II or III breast cancer	11	50.90 ± 8.60		448.2 ± 145.6	LLLT (PBM) – Comby 3 Terza Serie (ASA S.r.l., Italy)	Infrared laser; wavelengths 808 nm + 905 nm; average output at 905 nm: 24 mW (pulsed, 1–10, 000 Hz); max power at 808 nm: 500 mW (continuous/pulsed, 1–1500 Hz); energy density 2 J/cm²	3 sessions/week for 4 weeks (12 sessions); scanning over 144 cm²; ~20 min/session; supine position; non-contact scanning (distance 50 cm)	Axillary region of affected side (entire axilla, ~144 cm²)	10	51.30 ± 8.90	Sham active	Limb volume	Not reported	2, 4 weeks
Ridner SH, Poage-Hooper E, et al. (2013)a	USA	RCT, 3 arms	Stages II or III breast cancer	15	61.60 ± 9.90		Not reported	LLLT (PBM) – RianCorp LTU 904 (FDA-approved, Class 1)	Ga–As laser; wavelength 904 nm; Class 1; contact application; exposure 20–30 s/point; grid-based application	20 min/session; exposure controlled by timer; total sessions NR	Grid-defined treatment areas (affected upper limb)	8	63.90 ± 10.70	MLD	Limb volume Pain, Heaviness	Not reported	2, 4 weeks
Ridner SH, Poage-Hooper E, et al. (2013)b	USA	RCT, 3 arms	Stages II or III breast cancer	15	58.60 ± 11.00		Not reported	LLLT (PBM) – RianCorp LTU 904 (FDA-approved, Class 1)	Ga–As laser; wavelength 904 nm; Class 1; contact application; exposure 20–30 s/point; grid-based application	20 min/session; exposure controlled by timer; total sessions NR	Grid-defined treatment areas (affected upper limb)	8	63.90 ± 10.70	MLD	Limb volume Pain Heaviness	Not reported	2, 4 weeks
Selcuk Yilmaz S, Ayhan FF. (2023)	Turkey	RCT, 3 arms	Stages II or III breast cancer	15	55.30 ± 12.10		Not reported	LLLT (PBM) – (BTL-5000^®^, BTL Industries Ltd., UK)	Ga-Al-As laser; power density 30 mW/cm²; energy density 1.5 J/cm²; direct contact; grid technique	1 min/point; total 20min/session; LLLT followed by a multilayer bandaging; patient supine with arm abducted 90°	Axillary lymphatics (12 points); cubital/volar elbow lymphatics (8 points)	16	57.60 ± 9.50	MLD	Limb volume	Not reported	4, 8, 12 weeks
Storz MA, Gronwald B, et al. (2017)	Germany	RCT, 2 arms	>= 3 months	17	61.06 ± 9.66		Not reported	Cluster PBM – TIMELAS Vital (Schwa-medico, Germany)	Continuous-wave laser; wavelength 980 nm (NIR); 16 laser diodes; average power 40 mW/diode; total power 640 mW; spot size per diode 4.9 cm²; total beam area 78.54 cm²; energy per point 24 J; total energy 384 J; power density 8.14 mW/cm²; energy density 4.89 J/cm²	10 min/session (600 s); 2 sessions/week for 4 weeks (8 sessions total)	Axillary region (entire axilla)	19	59.37 ± 10.16	Sham active	Limb volume Pain, Handgrip strength, Quality of life	Not reported	4, 8, 12 weeks

MLD, Manual lymphatic drainage; PBM, Photobiomodulation; RCT, Randomized controlled trial; ROM, Range of motion; Baseline excess limb volume (mL): affected – unaffected. Excess limb volume was defined as affected minus unaffected limb volume and was reported in two trials; other studies were marked as not reported.

All interventions involved laser-based PBM, predominantly using low-intensity lasers, applied to the axillary lymphatic regions of the affected upper limb, although treatment parameters and irradiation areas differed among studies. Intervention duration ranged from 3 to 12 weeks, with treatment frequency varying from once daily to twice weekly. Regarding safety, only one study reported a single case of cellulitis, with no other serious adverse events observed, while the remaining studies did not provide explicit safety data (19). Notably, one included trial employed a three-arm design and was treated as two independent comparisons in this review, resulting in a total of ten comparisons included in the quantitative synthesis.

### 3.3 Risk of bias

**Figure 2:**
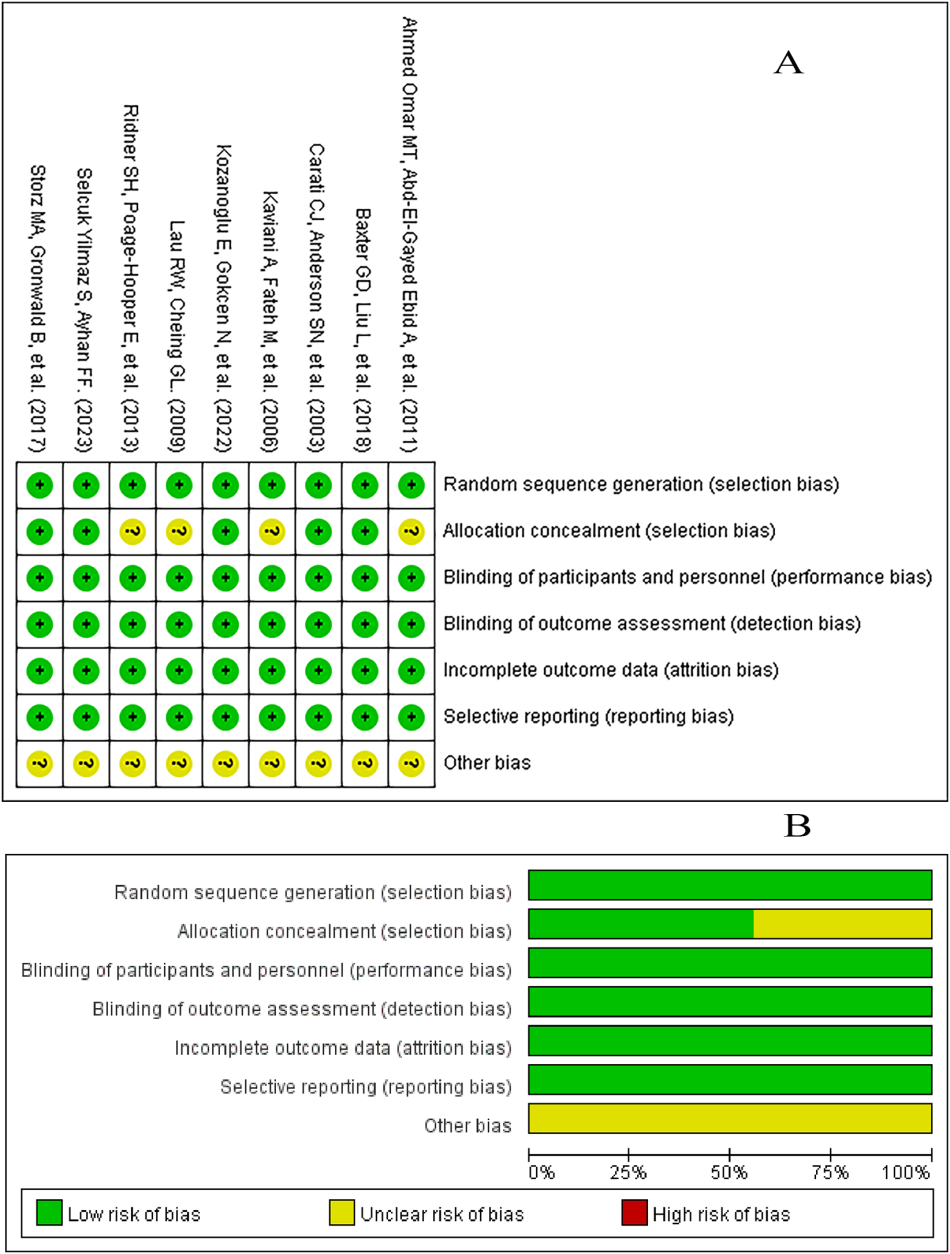
Summary and visualization of risk of bias assessment. **(A)** Risk of bias summary. “+” means low-risk bias; “?” means unclear risk bias; “-” means high-risk bias; **(B)** Deviation chart for risk of bias.

Two reviewers independently evaluated the nine RCTs using the Cochrane Risk of Bias 2 tool (**Figure 2**). Overall risk of bias across studies was low to moderate: most trials were judged as low risk of bias; however, four trials had unclear reporting of allocation concealment and were therefore rated as having some concerns in the randomization process domain. Although the majority of domains were low risk, these limitations could introduce selection bias and should be considered when interpreting the pooled results.

### 3.4 Synthesis of results

#### 3.4.1 Limb volume

**Figure 3:**
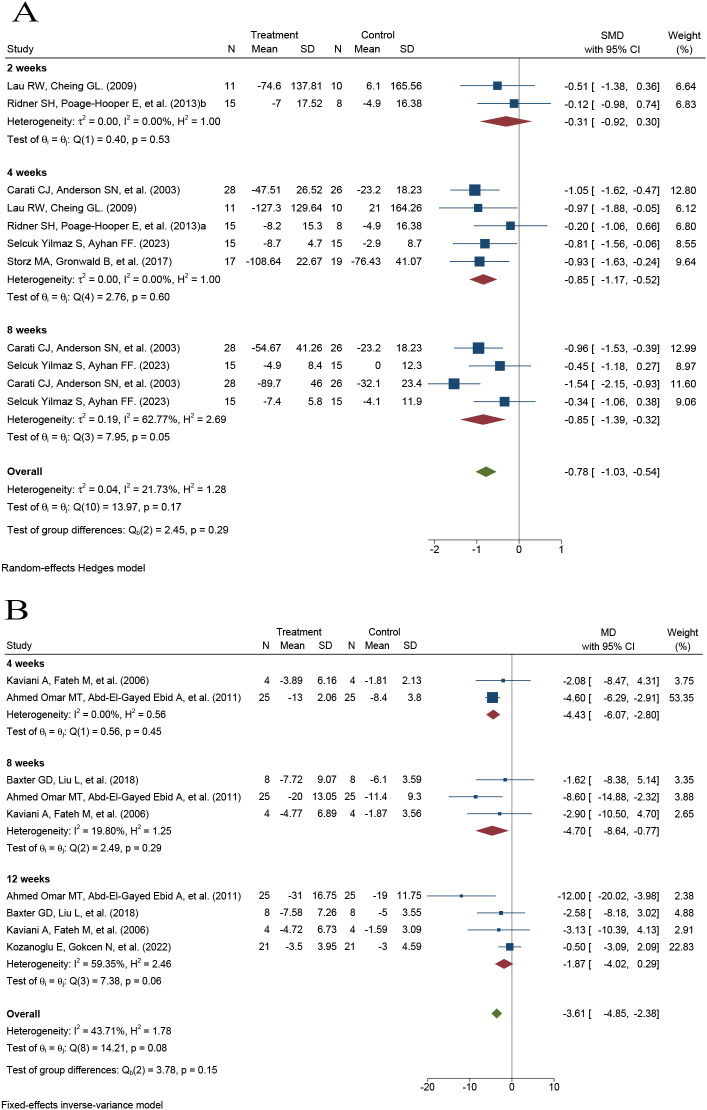
Forest plots showing the effects of photobiomodulation on limb volume and limb circumference in patients with breast cancer–related lymphedema. **(A)** Limb volume; **(B)** Limb circumference.

Six RCTs involving 187 participants were included in the analysis of limb volume. In accordance with the prespecified methodology, a random-effects model was applied. The pooled analysis showed that PBM therapy was associated with a significant reduction in limb volume compared with control interventions (SMD = −0.78, 95% CI −1.03 to −0.54; p < 0.001). The observed heterogeneity was low to moderate (I² = 22%, p = 0.17) (**Figure 3A**).

#### 3.4.2 Limb circumference

Four RCTs involving 116 participants were included in the meta-analysis of limb circumference. In accordance with the prespecified methodology, a fixed-effects model was applied. Compared with control interventions, PBM therapy significantly reduced limb circumference (MD = −3.61 cm, 95% CI −4.85 to −2.38; p < 0.001). Low to moderate heterogeneity was observed among studies (I² = 44%, p = 0.08) (**Figure 3B**).

#### 3.4.3 Hand grip strength

**Figure 4:**
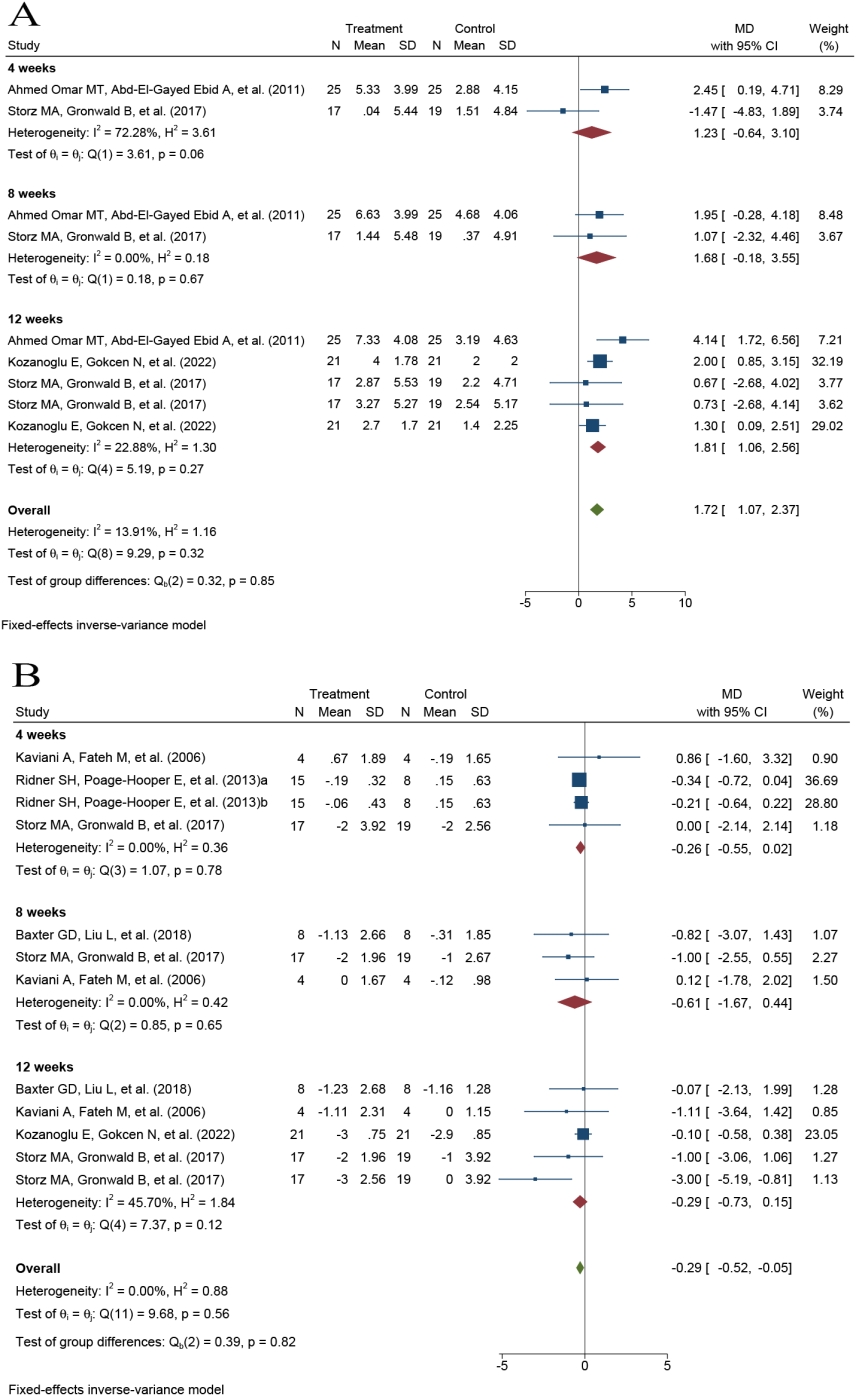
Forest plots of the effects of photobiomodulation on grip strength and pain in patients with breast cancer–related lymphedema. **(A)** Grip strength; **(B)** Pain.

Three RCTs, including 128 participants, were pooled to examine the effect of PBM on the grip strength of the affected upper limb. In line with the prespecified methodology, a fixed-effects model was applied. PBM therapy was associated with a significant increase in grip strength compared with control interventions (MD = 1.72 kg, 95% CI 1.07 to 2.37; p < 0.001). Low heterogeneity was observed across studies (I² = 14%, p = 0.32) (**Figure 4A**).

#### 3.4.4 Pain

Six RCTs involving 148 participants were pooled to evaluate the effect of PBM on pain in the affected upper limb. In accordance with the prespecified methodology, a fixed-effects model was used. PBM therapy was associated with a significant reduction in pain compared with control interventions (MD = −0.29, 95% CI −0.52 to −0.05; p < 0.001). No heterogeneity was detected among studies (I² = 0%, p = 0.56) (**Figure 4B**).

### 3.5 Adverse events

Reporting of adverse events varied across studies. Only one trial explicitly reported safety data, documenting a single case of cellulitis, whereas the remaining studies did not report adverse events. Adverse events were sought and extracted from all eligible trials; when not reported, this was recorded as “not reported” rather than assumed absent. The absence of reported events in these trials should not be interpreted as evidence of safety. Owing to insufficient and inconsistent reporting, a quantitative synthesis of safety outcomes was not feasible.

### 3.6 Sensitivity analyses

Sensitivity analyses were conducted to assess the robustness of the pooled results. For limb volume and circumference outcomes, leave-one-out analyses showed that removing any single study did not change the direction or statistical significance of the pooled effects. However, minor variations in effect size were observed.

### 3.7 GRADE evaluation of evidence

GRADE ratings and the Summary of Findings are summarized in **Table 2**, with full domain-specific evaluations provided in the accompanying source data. Evidence certainty for the primary outcomes ranged from low to moderate, driven mainly by downgrading for imprecision. This limitation largely reflected the small aggregated sample size (fewer than 400 participants), which constrained the precision of the pooled estimates. We did not downgrade for inconsistency because effect directions were generally consistent across trials, statistical heterogeneity was low to moderate (I² typically <50%), and sensitivity analyses did not materially change the pooled estimates. Although PBM parameters varied across studies, this protocol heterogeneity primarily limited identification of the optimal regimen rather than indicating substantial unexplained inconsistency in effect estimates.

**Table 2:** Summary of primary outcomes with GRADE evidence assessment.

Outcomes	No. of studies	No. of patients	Effect size (MD or SMD), [± 95% CI]	Anticipated absolute effects (95% CI)	P value	Heterogeneity assessment	Reason for downgrading
			Grade evaluation	Risk with photobiomodulation		I^2^ [p value]	
Limb Volume	6	187	SMD=-0.78	1.03 lower	P<0.001	I^2^ = 22%	Downgraded for imprecision due to the small total sample size (<400 participants)
			95% CI [-1.03 to -0.54]	(1.03 to 0.54 lower)		[P = 0.17]	
			⊕⊕⊕⊝				
			Moderate				
Limb Circumference (CM)	4	116	MD=-3.61	4.85 lower	P<0.001	I^2^ = 44%	Downgraded for imprecision due to the small total sample size (<400 participants)
			95% CI [-4.85 to -2.38]	(4.85 to 2.38 lower)		[P = 0.08]	
			⊕⊕⊕⊝				
			Moderate				
Grip strength (Kg)	3	128	MD=1.72	1.07 higher	P<0.001	I^2^ = 14%	Downgraded for imprecision due to the small total sample size (<400 participants)
			95% CI [1.07 to 2.37]	(1.07 higher to 2.37 higher)		[P = 0.32]	
			⊕⊕⊕⊝				
			Moderate				
Pain	6	148	MD=-0.29	0.52 lower	P<0.001	I^2^ = 0%	Downgraded for imprecision due to the small total sample size (<400 participants)
			95% CI [-0.52 to -0.05]	(0.52 lower to 0.05 lower)		[P = 0.56]	
			⊕⊕⊕⊝				

GRADE: Grading of Recommendations, Assessment, Development, and Evaluations; SMD: Standardized mean difference; MD: Mean difference. We did not downgrade for inconsistency because heterogeneity was low–moderate and effects were directionally consistent; sensitivity analyses were robust.

## 4 Discussion

This systematic review and meta-analysis synthesizes randomized evidence to clarify the clinical role of PBM in the management of BCRL. PBM was consistently associated with reductions in upper-limb swelling, reflected by decreased limb volume and circumference, along with improvements in grip strength and pain. These effects showed low between-study heterogeneity and remained stable in sensitivity analyses, supporting the robustness of the pooled estimates. However, evidence certainty ranged from low to moderate under GRADE, mainly due to imprecision from limited cumulative sample sizes, and a subset of trials had some concerns in the randomization process (e.g., unclear allocation concealment), warranting cautious interpretation.

When edema-related outcomes were examined over time, PBM appeared to show a time-dependent pattern. Early effects were not evident at approximately 2 weeks, whereas reductions in limb volume and circumference became apparent from around 4 weeks and were sustained through 8 weeks. This suggests that PBM may require cumulative exposure before clinically detectable changes in swelling occur, which is biologically plausible given the time needed for lymphatic functional adaptation, modulation of local inflammation, and improvements in tissue compliance (25, 26). The lack of statistical significance at 12 weeks should be interpreted cautiously, as it may reflect fewer contributing studies, smaller cumulative sample sizes, and greater variability in treatment parameters and follow-up timing rather than a true waning of effect. Taken together, these findings suggest that the most consistent anti-edematous effects may be observed within an intermediate treatment window of approximately 4–8 weeks, while durability beyond this period requires confirmation in larger trials with longer follow-up.

Clinically, the interpretability of edema reduction is enhanced when changes are anchored to baseline severity and commonly used diagnostic thresholds in BCRL. In practice, BCRL is often defined or staged using interlimb differences, for example, an interlimb volume difference of ≥10%, alongside circumference-based criteria in some settings (27). However, baseline edema severity was reported heterogeneously across the included trials, and only two studies provided baseline excess limb volume (affected minus unaffected), which precluded a consistent cross-trial estimation of percentage reduction relative to baseline. Notwithstanding, the available baseline data (mean excess volumes of approximately 448–888 mL) indicate clinically relevant swelling in at least a subset of participants. Interpretation should also consider the minimal clinically important difference (MCID): to date, no universally accepted MCID thresholds have been established for limb volume or circumference in BCRL, and reported benchmarks vary by measurement technique, baseline severity, and analytic approach. In the absence of established MCIDs, greater weight should be placed on the coherence of effects across complementary domains and on patient-centered responder metrics. In this review, improvements in swelling outcomes were accompanied by favorable changes in pain and functional performance, supporting the possibility of patient-relevant benefit beyond statistical significance (28). Future trials of PBM in BCRL should therefore standardize baseline reporting and prespecify relative (% baseline) change and responder analyses (e.g., the proportion improving below clinically meaningful thresholds such as <10% interlimb difference), together with longer follow-up, to strengthen clinical interpretability and better define durability and optimal treatment duration (29, 30).

In contrast to swelling-related endpoints, the time pattern of effects on grip strength and pain appears more delayed, suggesting that functional and symptomatic benefits may require longer exposure and/or follow-up to become clinically detectable (31). This is clinically plausible because grip strength is a composite indicator of upper-limb capacity and depends not only on tissue status but also on pain inhibition, joint mobility, neuromuscular performance, and patients’ engagement in daily activities or rehabilitation exercises. Likewise, pain relief may reflect gradual changes in local inflammation and mechanosensitivity, reduced tissue pressure, and progressive adaptation to movement, rather than an immediate analgesic effect (32, 33). As a result, improvements in function and symptoms may emerge downstream of earlier reductions in edema and tissue stiffness, consistent with a “cascade” model in which initial tissue-level changes enable later performance gains. This interpretation aligns with prior clinical observations that PBM-related improvements in edema markers tend to precede more gradual gains in functional performance and symptom relief (34, 35). From a translational and trial-design perspective, these findings underscore the need for sufficient treatment duration and adequately timed follow-up assessments when evaluating PBM effects on function- and pain-related outcomes; otherwise, short-term evaluations may underestimate delayed but clinically relevant benefits.

PBM may exert therapeutic effects in BCRL through multiple complementary biological pathways. Light absorption by cytochrome c oxidase in the mitochondrial membrane may enhance adenosine triphosphate production and cellular oxygen utilization, thereby supporting metabolic activity and tissue repair (36, 37). At the lymphatic level, PBM may stimulate lymphatic endothelial cell function, potentially promoting lymphangiogenesis as well as rhythmic contraction and dilation of lymphatic vessels, while improving tissue compliance and local fluid dynamics to facilitate edema resorption (38, 39). PBM has also been reported to exert anti-inflammatory and anti-edematous effects, potentially through modulation of prostaglandin I_2_ levels, leading to reduced platelet aggregation, vasodilation, and improved tissue oxygenation (40). In addition, PBM may influence macrophage and leukocyte activity, which could aid clearance of inflammatory mediators and reduce the risk of skin infections (41, 42). Together, these mechanisms provide a plausible biological basis for the reductions in limb volume and circumference observed in this study. As tissue pressure and inflammation decrease, pain perception may be alleviated and mechanical conditions for movement improved, which in turn may facilitate downstream functional gains such as improved grip strength. Overall, PBM may influence morphological, functional, and symptomatic outcomes in BCRL through an integrated pathway involving enhanced cellular energy metabolism, improved lymphatic drainage, modulation of inflammation, and optimization of the local tissue environment. To strengthen translational inference, future PBM trials in BCRL should incorporate standardized immune/inflammation biomarker monitoring, such as cytokine profiles (e.g., IL-6, TNF-α, IL-1β, IL-10, TGF-β), macrophage polarization markers (e.g., M1/M2-related markers), and lymphatic endothelial inflammatory/lymphangiogenic signaling (e.g., VEGF-C/VEGFR-3 and related endothelial activation markers), alongside clinical volume and symptom outcomes.

Overall, the certainty of evidence ranged from low to moderate, mainly due to imprecision from small pooled sample sizes. Nevertheless, key outcomes showed consistent effect directions, low heterogeneity, and stable sensitivity analyses, supporting the internal reliability of the findings. These limitations reflect study scale and methodological variability rather than uncertainty in effect direction, suggesting that the results provide cautious support for PBM as an adjunctive option for BCRL, while highlighting the need for larger, standardized trials with longer follow-up.

## 5 Limitations

This review has several limitations. The limited number of trials and small cumulative sample size reduced statistical precision for some outcomes. Although statistical heterogeneity was low for several outcomes, there was substantial clinical heterogeneity in PBM protocols across studies (e.g., wavelength, energy density/dose, treatment duration, and application frequency), which may have influenced pooled estimates and limited identification of optimal treatment parameters. In addition, unclear or incomplete reporting of randomization procedures, allocation concealment, and adverse events in some trials introduces methodological uncertainty and restricts conclusions regarding PBM safety, particularly because safety outcomes could not be synthesized quantitatively. Time-stratified subgroup analyses were conducted for each outcome; however, subgroup analyses by overall treatment duration or specific PBM parameter ranges were not feasible because too few studies were available within comparable categories, and parameter reporting was inconsistent. Finally, the lack of an established minimal clinically important difference for limb volume and circumference limits the clinical interpretation of statistically significant results.

## 6 Conclusion

This systematic review and meta-analysis suggest that PBM may offer clinical benefits in the management of BCRL. PBM was associated with improvements in upper-limb swelling and with delayed gains in grip strength and pain relief, indicating a time-dependent treatment effect. Although the certainty of evidence was low to moderate, PBM may be considered as an adjunctive intervention; however, conclusions regarding safety are limited by sparse and inconsistent adverse-event reporting, and its optimal treatment parameters and long-term effectiveness require confirmation in larger, well-designed trials.

## References

[1] Bizuayehu HM Dadi AF Hassen TA Ketema DB Ahmed KY Kassa ZY . Global burden of 34 cancers among women in 2020 and projections to 2040: Population-based data from 185 countries/territories. Int J Cancer. (2024) 154:1377–93. doi: 10.1002/ijc.34809. PMID: 38059753

[2] Luo C Li N Lu B Cai J Lu M Zhang Y . Global and regional trends in incidence and mortality of female breast cancer and associated factors at national level in 2000 to 2019. Chin Med J. (2022) 135:42–51. doi: 10.1097/cm9.0000000000001814. PMID: 34593698 PMC8850868

[3] Jørgensen MG Toyserkani NM Hansen FG Bygum A Sørensen JA . The impact of lymphedema on health-related quality of life up to 10 years after breast cancer treatment. NPJ Breast Cancer. (2021) 7:70. 34075045 10.1038/s41523-021-00276-y PMC8169644

[4] Bakar Y . Managing Side Effects of Breast Cancer Treatment. Cham: Springer (2025). p. 99–114.

[5] Sharifi N Ahmad S . Breast cancer-related lymphedema: a critical review on recent progress. Surg Oncol. (2024) 56:102124. doi: 10.1016/j.suronc.2024.102124. PMID: 39208532

[6] He L Qu H Wu Q Song Y . Lymphedema in survivors of breast cancer. Oncol Lett. (2020) 19:2085–96. doi: 10.3892/ol.2020.11307. PMID: 32194706 PMC7039097

[7] Deng C Wu Z Cai Z Zheng X Tang C . Conservative medical intervention as a complement to CDT for BCRL therapy: a systematic review and meta-analysis of randomized controlled trials. Front Oncol. (2024) 14:1361128. doi: 10.3389/fonc.2024.1361128. PMID: 38737896 PMC11082302

[8] Foeldi E . Peripheral Lymphedema: Pathophysiology, Modern Diagnosis and Management. Singapore: Springer (2021). p. 189–94.

[9] Ren Y Ge R Yang C Tan Y Song H Liu R . Efficacy of complex decongestive therapy in managing limb swelling, pain, and enhancing functional recovery after arthroscopic reconstruction of anterior cruciate ligament. Appl Nurs Res. (2025) 82:151915. doi: 10.1016/j.apnr.2025.151915. PMID: 40086932

[10] Gultekin SC Karadibak D . Cancer Metastasis, Management and Complications: An Interdisciplinary Approach. Cham: Springer (2024). p. 315–54.

[11] Storz MA Gronwald B Gottschling S Schöpe J Mavrova R Baum S . Photobiomodulation therapy in breast cancer-related lymphedema: a randomized placebo-controlled trial. Photodermatol Photoimmunol Photomedicine. (2017) 33:32–40. doi: 10.1111/phpp.12284. PMID: 27943450

[12] Ridner SH Poage-Hooper E Kanar C Doersam JK Bond SM Dietrich MS . A pilot randomized trial evaluating low-level laser therapy as an alternative treatment to manual lymphatic drainage for breast cancer-related lymphedema. Oncol Nurs Forum. (2013) 40:383–93. doi: 10.1188/13.onf.383-393. PMID: 23803270 PMC3887507

[13] Barolet AC Villarreal AM Jfri A Litvinov IV Barolet D . Low-intensity visible and near-infrared light-induced cell signaling pathways in the skin: a comprehensive review. Photobiomodulation Photomed Laser Surg. (2023) 41:147–66. doi: 10.1089/photob.2022.0127. PMID: 37074309

[14] da Silva TG Ribeiro RS Mencalha AL de Souza Fonseca A . Photobiomodulation at molecular, cellular, and systemic levels. Lasers Med Sci. (2023) 38:136. doi: 10.1007/s10103-023-03801-6. PMID: 37310556

[15] Deka T Kalita P Goswami K Mattaparthi VSK . Advanced Biophysical Techniques in Biosciences. Cham: Springer (2025). p. 301–25.

[16] Glass GE . Photobiomodulation: A review of the molecular evidence for low level light therapy. J Plastic Reconstructive Aesthetic Surg. (2021) 74:1050–60. doi: 10.1016/j.bjps.2020.12.059. PMID: 33436333

[17] Sterne JA Sutton AJ Ioannidis JP Terrin N Jones DR Lau J . Recommendations for examining and interpreting funnel plot asymmetry in meta-analyses of randomised controlled trials. BMJ. (2011) 343:d4002-4013. doi: 10.1136/bmj.d4002. PMID: 21784880

[18] Ahmed Omar MT Abd-El-Gayed Ebid A El Morsy AM . Treatment of post-mastectomy lymphedema with laser therapy: double blind placebo control randomized study. J Surg Res. (2011) 165:82–90. doi: 10.1016/j.jss.2010.03.050. PMID: 20538293

[19] Baxter GD Liu L Tumilty S Petrich S Chapple C Anders JJ . Low level laser therapy for the management of breast cancer-related lymphedema: a randomized controlled feasibility study. Lasers Surg Med. (2018) 50:924–32. doi: 10.1002/lsm.22947. PMID: 29851090

[20] Carati CJ Anderson SN Gannon BJ Piller NB . Treatment of postmastectomy lymphedema with low-level laser therapy: a double blind, placebo-controlled trial. Cancer. (2003) 98:1114–22. 10.1002/cncr.11641 12973834

[21] Kaviani A Fateh M Yousefi-Nooraie R Alinagi-Zadeh MR Ataie-Fashtami L . Low-level laser therapy in management of postmastectomy lymphedema. Lasers Med Sci. (2006) 21:90–4. doi: 10.1007/s10103-006-0380-3. PMID: 16673054

[22] Kozanoglu E Gokcen N Basaran S Paydas S . Long-Term Effectiveness of Combined Intermittent Pneumatic Compression Plus Low-Level Laser Therapy in Patients with Postmastectomy Lymphedema: a Randomized Controlled Trial. Lymphatic Res Biol. (2022) 20:175–84. doi: 10.1089/lrb.2020.0132. PMID: 33826415

[23] Lau RW Cheing GL . Managing postmastectomy lymphedema with low-level laser therapy. Photomed Laser Surg. (2009) 27:763–9. doi: 10.1089/pho.2008.2330. PMID: 19878027

[24] Selcuk Yilmaz S Ayhan FF . The Randomized Controlled Study of Low-Level Laser Therapy, Kinesio-Taping and Manual Lymphatic Drainage in Patients With Stage II Breast Cancer-Related Lymphedema. Eur J Breast Health. (2023) 19:34–44. doi: 10.4274/ejbh.galenos.2022.2022-6-4. PMID: 36605467 PMC9806938

[25] Reis L Silva T Januário PG . Photobiomodulation Associated or Not with Other Therapeutic Techniques in the Treatment of Post-Breast Cancer Lymphedema: Literature Systematic Review. Rev Bras Cancerologia. (2025) 71:e-255249. doi: 10.17504/protocols.io.n92ldpjkol5b/v1. PMID: 41033768

[26] Chiu S-T Lai U-H Huang Y-C Leong C-P Chen P-C . Effect of various photobiomodulation regimens on breast cancer-related lymphedema: A systematic review and meta-analysis. Lasers Med Sci. (2023) 39:11. doi: 10.1007/s10103-023-03959-z. PMID: 38129368

[27] Wagner BD Barrio AV Coriddi MR Rubin J Boe LA Shammas RL . Redefining the Diagnostic Threshold for Breast Cancer-related Lymphedema. Ann Surg. (2025) 38:10.1097. doi: 10.1097/sla.0000000000006983. PMID: 41220050

[28] Min J Park Y . Clinical Applications of Photobiomodulation Therapy in the Management of Breast Cancer-related Lymphedema. Med Lasers Engineering Basic Research Clin Appl. (2021) 10:189–94. doi: 10.25289/ml.2021.10.4.189

[29] Hussein HM Gabr AMM Fadulelmulla IA Aldemery AA Ragab MMM Mohamed AR . Impact of low-level laser therapy on upper limb lymphoedema secondary to breast cancer: a systematic review and meta-analysis. Arch Med Sci. (2025) 21:889–96. doi: 10.5114/aoms/186874. PMID: 40741271 PMC12305524

[30] Mahmood D Ahmad A Sharif F Arslan SA . Clinical application of low-level laser therapy (Photo-biomodulation therapy) in the management of breast cancer-related lymphedema: a systematic review. BMC Cancer. (2022) 22:937. doi: 10.1186/s12885-022-10021-8. PMID: 36042421 PMC9426030

[31] Frankowski DW Ferrucci L Arany PR Bowers D Eells JT Gonzalez-Lima F . Light buckets and laser beams: mechanisms and applications of photobiomodulation (PBM) therapy. GeroScience. (2025) 47(3):2777–89. doi: 10.1007/s11357-025-01505-z. PMID: 39826026 PMC12181550

[32] Merkle SL Sluka KA Frey-Law LA . The interaction between pain and movement. J Handb Ther. (2020) 33:60–6. doi: 10.1016/j.jht.2018.05.001. PMID: 30025839 PMC6335190

[33] Chen Q Heinricher MM . Plasticity in the link between pain-transmitting and pain-modulating systems in acute and persistent inflammation. J Neurosci. (2019) 39:2065–79. doi: 10.1523/jneurosci.2552-18.2019. PMID: 30651329 PMC6507088

[34] Labib CM Mowafy ZME Gaber AAS Ali KM . Post-Mastectomy Shoulder Pain and Lymphedema Responses to Ga-As Laser Versus Microcurrent Electrical Stimulation. J Advanced Zoology. (2023) 44:762–808. doi: 10.17762/jaz.v44is7.2808

[35] Robijns J Lodewijckx J Claes M Lenaerts M Van Bever L Claes S . A long‐term follow‐up of early breast cancer patients treated with photobiomodulation during conventional fractionation radiotherapy in the prevention of acute radiation dermatitis. Lasers Surg Med. (2022) 54:1261–8. doi: 10.1002/lsm.23608. PMID: 36183377

[36] Sommer AP . Mitochondrial cytochrome c oxidase is not the primary acceptor for near infrared light—it is mitochondrial bound water: the principles of low-level light therapy. Ann Trans Med. (2019) 7:S13. doi: 10.21037/atm.2019.01.43. PMID: 31032294 PMC6462613

[37] Hamblin MR Liebert A . Photobiomodulation therapy mechanisms beyond cytochrome c oxidase. Photobiomodulation Photomedicine Laser Surg. (2022) 40:75–7. doi: 10.1089/photob.2021.0119. PMID: 34818111

[38] Jibawi A . Modelling and quantifying the impact of photobiomodulation (PBM) on biological processes relevant to lymphangiogenesis, anti-inflammation, and tissue regeneration processes: results from a meta-analysis and a neural network modelling. Dermatol J Cosmet Laser Ther. (2024) 3(1):1–9.

[39] Semyachkina-Glushkovskaya O Abdurashitov A Dubrovsky A Klimova M Agranovich I Terskov A . Photobiomodulation of lymphatic drainage and clearance: perspective strategy for augmentation of meningeal lymphatic functions. BioMed Opt Express. (2020) 11:725–34. doi: 10.1364/boe.383390. PMID: 32206394 PMC7041454

[40] Sukanya D Upasana L Deepak T Abhinethra M Choudary S . Determination of effectiveness of photobiomodulation in the treatment of oral submucous fibrosis. J Pharm Bioallied Sci. (2022) 14:S475–8. doi: 10.4103/jpbs.jpbs_673_21. PMID: 36110778 PMC9469282

[41] Woo K . Therapeutic implications of photobiomodulation application on immune cells. Med Lasers Engineering Basic Research Clin Appl. (2025) 14:119–26. doi: 10.25289/ml.25.023

[42] Courtois E Bouleftour W Guy J-B Louati S Bensadoun R-J Rodriguez-Lafrasse C . Mechanisms of PhotoBioModulation (PBM) focused on oral mucositis prevention and treatment: a scoping review. BMC Oral Health. (2021) 21:220. doi: 10.1186/s12903-021-01574-4. PMID: 33926421 PMC8086292

