# Supplementary material for: Effects of photobiomodulation therapy on upper limb lymphedema secondary to breast cancer: a systematic review and meta-analysis
Source: Front Oncol. 2026 Mar 23;16:1802643. doi: 10.3389/fonc.2026.1802643 (PMC13050686; doi:10.3389/fonc.2026.1802643)
Supplement: Supplementary file 1 [file Table1.docx]

**Supplementary Table 1.** Search Strategy.

#1 Breast Neoplasms **[Mesh]**

#2 (breast And neoplasm*) **[Title/Abstract]**

#3 (breast And carcinoma*) **[Title/Abstract]**

#4 (breast and cancer*) **[Title/Abstract]**

#5 (breast And tumour*) **[Title/Abstract]**

#6 (breast And tumor*) **[Title/Abstract]**

#7 #1 OR #2 OR #3 OR #4 OR #5 OR #6

#8 Lymphedema **[Mesh]**

#9 lymphoedema* OR lymphedema* OR (lymphatic oedema*) OR (lymphatic edema*) OR oedema* OR edema* OR (arm oedema) OR (arm edema) OR (upper extremity oedema) OR (upper extremity edema) OR (lymphoedemic) OR (lymphedemic)

#10 #8 OR #9

#11 Low‐Level Light Therapy **[Mesh]**

#12 (low level light) **[Title/Abstract]**

#13 ((low level **OR** low power **OR** low energy **OR** low intensity) **And** laser*) **[Title/Abstract]**

#14 (light based **And** technolog*) **[Title/Abstract]**

#15 (photobiomodulat* **OR** PBM **OR** photomodulat*) **[Title/Abstract]**

#16 (near-infrared laser) **OR** (transcranial laser) **[Title/Abstract]**

#17 phototherapy **[Title/Abstract]**

#18 (laser* **And** (irradiat* **OR** phototherapy* **OR** biostimulat*)) **[Title/Abstract]**

#19 (phototherapy* **And** infrared) **[Title/Abstract]**

#20 #11 OR #12 OR #13 OR #14 OR #15 OR #16 OR #17 OR #18 OR #19

#21 (randomized controlled trial[pt] OR controlled clinical trial[pt] OR randomized[tiab] OR placebo[tiab] OR clinical trials as topic[mesh:noexp] OR randomly[tiab] OR trial[ti]) NOT (animals [mh] NOT (humans [mh] AND animals[mh]))

#22 #7 And #10 And #20 And #21
